# Viral Infections in Special Populations: Key Findings and Perspectives from Special Issue

**DOI:** 10.3390/v17060806

**Published:** 2025-05-31

**Authors:** Eleni Polyzou, Karolina Akinosoglou

**Affiliations:** 1 Department of Medicine, University of Patras, 26504 Rio, Greece; polyzou.el@gmail.com; 2 Department of Internal Medicine, University General Hospital of Patras, 26504 Rio, Greece; 3 Division of Infectious Diseases, University General Hospital of Patras, 26504 Rio, Greece

Viral infections pose a significant epidemiological burden worldwide. While understanding the core epidemiological factors—such as viral characteristics, host immunity, and transmission dynamics—provides a foundation for evidence-based prevention strategies, the situation becomes more nuanced when considering special populations. These groups include individuals living with HIV (PLWHIV), those who are pregnant or lactating, the immunocompromised (IC), elderly, pediatric and adolescent patients, and those with hematologic disorders, liver cirrhosis, obesity, or undergoing renal replacement therapy. They are not only more susceptible to infection but also frequently present with atypical disease manifestations, experience delayed diagnoses, and face more complicated treatment courses, with many cases progressing to critical illness requiring intensive care. This Special Issue features eight articles that offer novel insights into the epidemiology, clinical outcomes, viral evolution, and co-infection dynamics affecting these vulnerable populations.

COVID-19 stands as one of the most significant paradigms of viral disease in modern history, illustrating the profound global impact a novel pathogen can have on public health, economies, and societies. As defined by the World Health Organization (WHO), COVID-19 is a global outbreak caused by severe acute respiratory syndrome coronavirus 2 (SARS-CoV-2) [1]. Throughout the pandemic, the case fatality rate (CFR) varied significantly across SARS-CoV-2 variants, reflecting changes in both viral characteristics and population immunity over time [2]. In this context, Livieratos et al. investigated the impact of SARS-CoV-2 variants on clinical outcomes—such as hospitalization, ICU admission, and mortality—across the following four special populations: immunocompromised individuals, PLWHIV, pediatric patients, and those with chronic liver disease (CLD) (https://www.mdpi.com/1999-4915/16/8/1222, accessed on 9 May 2025). Based on the analysis of 23 relevant articles, the review found that earlier variants like Alpha and Delta were associated with more severe outcomes, while Omicron—though milder overall—continued to pose a serious threat to vulnerable groups.

Viral persistence [3], coupled with high mutation rates [4], has been linked to increased mortality rates and worsened clinical outcomes in IC patients. However, the findings of a study by Wouters et al. challenge some of the conclusions drawn from earlier case reports (https://www.mdpi.com/1999-4915/16/9/1436, accessed on 9 May 2025). Specifically, the researchers investigated how prolonged SARS-CoV-2 infections in two IC patients drove viral evolution by analyzing live virus isolates. They examined replication dynamics, syncytia formation, immune escape potential, and genetic mutations, especially in the Spike protein. Interestingly, one patient’s virus showed replication attenuation without developing immune escape mutations, whereas the virus from the other patient retained replication fitness and evolved immune escape traits along with enhanced cell–cell fusion. These contrasting findings emphasize that viral adaptation in IC patients is shaped by host-specific immune environments, suggesting that disease outcomes in such individuals may be more heterogeneous than previously assumed.

More importantly, the COVID-19 pandemic likely contributed to delayed diagnoses of critical conditions such as HIV and cancer, often resulting in patients presenting at more advanced stages [5]. Padurariu-Covit et al. highlighted how the pandemic led to late diagnoses of such conditions and the increased complexity of managing co-infections with SARS-CoV-2. They presented a case report of a 57-year-old patient with advanced-stage Hodgkin lymphoma, HIV infection, and COVID-19 pneumonia (https://www.mdpi.com/1999-4915/17/3/404, accessed on 9 May 2025).

Finally, Bartlett et al. pointed out that individuals with heritable connective tissue diseases were at an increased risk not only for severe COVID-19 but also for developing post-acute sequelae of SARS-CoV-2 infection (https://www.mdpi.com/1999-4915/16/3/461, accessed on 9 May 2025), aligning with findings from other studies on special populations [6,7].

Other articles in this Issue provide valuable information about viral infections in PLWHIV. In a national cross-sectional pilot study conducted in Greece, Schinas et al. found a low prevalence of Hepatitis D virus (HDV) among PLWHIV with chronic Hepatitis B infection. Only one individual (3%) tested positive for anti-HDV antibodies, and none for HDV RNA, suggesting a lower regional burden compared to global estimates (https://www.mdpi.com/1999-4915/16/7/1044, accessed on 9 May 2025). This finding is intriguing, as other studies have reported higher HDV prevalence rates in PLWHIV co-infected with chronic Hepatitis B [8,9]. Due to their immunocompromised status, PLWHIV are also at an increased risk of infections caused by special or atypical pathogens. A specific example is Human Herpes Virus 6 (HHV-6), which is frequently associated with encephalitis in IC individuals [10,11,12]. In a meta-analysis, Kostare et al. reported an HHV6 prevalence of 8% among PLWHIV, based on molecular diagnostic methods, highlighting its relevance as a significant opportunistic infection in this population (https://www.mdpi.com/1999-4915/17/4/531, accessed on 9 May 2025).

When examining cases involving significantly immunocompromised individuals, it is important to remain vigilant for opportunistic infections, which may pose minimal risk in healthy individuals but can lead to serious disease in those who are immunosuppressed [13]. Of particular interest is the frequent co-occurrence of opportunistic infections with other endemic pathogens, which can complicate clinical management and hinder accurate diagnosis due to overlapping symptoms and shared inflammatory responses [14]. In their study, Jett et al. found that 92% of patients infected with Herpes Virus 8 (HHV-8) were co-infected with both HIV and Epstein–Barr Virus (EBV), identifying a total of 43 co-infections. These findings support the previously discussed observations and underscore the importance of comprehensive diagnostic testing (https://www.mdpi.com/1999-4915/17/4/460, accessed on 9 May 2025).

Finally, refugee populations are considered particularly vulnerable to infections due to poor living conditions, insufficient disease prevention measures, and limited access to healthcare services [15]. A systematic review and meta-analysis of Marinho et al. (https://www.mdpi.com/1999-4915/16/10/1526, accessed on 9 May 2025) reported pooled global prevalence rates of Human T-lymphotropic virus 1 (HTLV-1) and Human T-lymphotropic virus 2 (HTLV-2) infections among immigrants and refugees at 1.28% and 0.11%, respectively. The study also revealed a significant regional variation of HTLV-1 prevalence, emphasizing the need for targeted public health interventions in non-endemic countries that receive refugees.

Overall, this Special Issue provides significant insights into how viral infections continue to pose a major global health threat, particularly for vulnerable populations who face increased risks of severe disease, complex diagnoses, and challenging treatment options. Understanding the intricate relationships between host immunity, viral evolution, and transmission dynamics is critical for developing more effective prevention and management strategies tailored to these high-risk groups.

Looking ahead, more comprehensive research is needed to better understand the unique immune responses and viral adaptations within vulnerable populations. Additionally, enhanced surveillance and diagnostic capabilities can facilitate the early detection of co-infections, thereby contributing to better clinical outcomes.
